# Two-stage hybrid treatment strategy for an adult patient with aortic arch coarctation, poststenotic aneurysm, and hypoplastic left subclavian artery: A case report

**DOI:** 10.1097/MD.0000000000008618

**Published:** 2017-12-01

**Authors:** Xiao-Bo Pu, Shi-Jian Chen, Mao Chen, Yuan Feng

**Affiliations:** Department of Cardiology, West China Hospital, Sichuan University, Chengdu, Sichuan, China.

**Keywords:** coarctation of aorta, extra-anatomic bypass, hybrid treatment strategy, muscular ventricular septal defect occluders, poststenotic aneurysm

## Abstract

**Rationale:**

Coarctation of aorta in adulthood is usually complicated by other cardiovascular anomalies, posing great technical challenge for intervention.

**Patient concerns:**

Here, we report an extremely rare case of aortic arch coarctation combined with a poststenotic biloculated calcified aneurysm and hypoplastic left subclavian artery.

**Interventions:**

First, an extra-anatomic bypass was established, along with narrowing of aorta just proximal and distal to the aneurysm. While the bypass graft significantly relieved trans-coarctation gradient, the latter procedure decreased intra-aneurysm pressure and created landing zones for aneurysm occlusion. Six months later, 2 muscular ventricular septal defect occluders were deployed at the proximal and distal orifice of the aneurysm.

**Outcomes:**

Follow-up computed tomography angiography confirmed the absence of contrast leakage into aneurysm.

**Conclusions:**

A 2-stage hybrid approach described here appears to be feasible, safe, and associated with favorable clinical outcomes in the treatment of complicated aortic coarctation and poststenotic aneurysm.

## 1 Introduction

Coarctation of the aorta is a common congenital heart disease that may go undetected well into adulthood. Sometimes, coarctation in adulthood is accompanied by congenital and acquired vascular pathology that may require surgical intervention. The management of an adult patient with aortic coarctation and other vascular anomalies poses a great technical challenge. We hereby present a rare case with the combination of aortic anomalies including an aortic arch coarctation, a poststenotic biloculated calcified aneurysm, and a hypoplastic left subclavian artery (LSA), which was successfully treated with a 2-stage hybrid approach.

## 2 Case presentation

Informed consent was obtained from the individual participant in the study.

**Figure 1:**
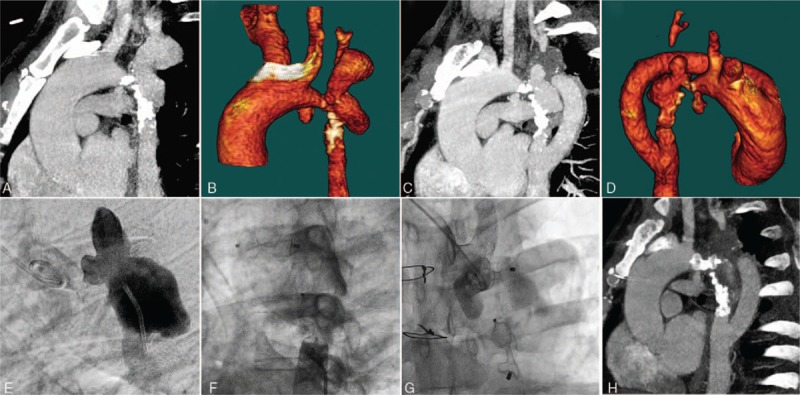
(A and B) Preoperative CTA shows an aortic arch coarctation located between left common carotid artery and left subclavian artery with a poststenotic biloculated calcified aneurysm. (C and D) Postoperative CTA shows patency of the extra-anatomic bypass graft and the aneurysm. (E) Angiography in the aneurysm shows the diameter of the proximal and distal tract connecting to the aneurysm was measured at 5 and 7 mm. (F and G) Angiography after occluders deployment distal to the aneurysm and proximal to the aneurysm shows that the aneurysm was completely isolated from the aortic cavity. (H) CTA at 3-month follow-up confirmed the absence of contrast leakage into the aneurysm and patency of the bypass graft. CTA = computed tomography angiography.

A 35-year-old man (body weight: 100 kg) was admitted to our center complaining of headache and chest discomfort. Physical examination revealed high blood pressure (184/88 mm Hg) in the right arm and weak lower extremity and left arm pulses. Echocardiography and computed tomography (CT) angiography demonstrated an aortic arch coarctation with a minimal diameter of 5 mm. The coarctation was located between the left common carotid artery and the LSA, with a large collateral circulation and a large biloculated calcified aneurysm just distal to the coarctation. The LSA arose from the upper locule of the aneurysm with an upper-to-lower flow direction (Fig. 1A and B). Coronary angiography and CT cerebral angiography are unremarkable.

Anatomical surgical aortoplasty or percutaneous transluminal aortoplasty for the complex lesion between the aortic arch and descending aorta would be a difficult as well as high-risk procedure. Therefore, the team decided to treat the patient via a 2-stage hybrid approach, with extra-anatomic bypass grafting performed first, followed by subsequent transluminal occlusion of the aneurysmal orifice several months later.

The first-stage procedure was performed under general anesthesia via a left thoracotomy without cardiopulmonary bypass or deep hypothermic circulatory arrest. A bypass between the ascending aorta and the descending aorta was established using a 16-mm Gore-Tex tube. Arterial ligation was performed at the aorta just proximal to the coarctation, at the descending aorta distal to the aneurysm, and at the origin of the LSA. Postoperative recovery was uneventful, with a decreased blood pressure difference between the right arm and lower limbs (preoperative and postoperative systolic blood pressure difference is 46 and 8 mm Hg, respectively). The patient was discharged 8 days after the operation in good condition.

Six months later, the patient was readmitted for transluminal occlusion of the aneurysmal orifice. Preoperative CT aortography showed persistent flow of contrast into the aneurysm sac (Fig. 1C and D). Right femoral and radial arteries were accessed with 6F sheaths. On aortography, the diameter of the proximal and distal tract connecting to the aneurysm was measured at 5 and 7 mm, respectively (Fig. 1E). A VER catheter over a hydrophilic guide wire was negotiated into the aneurysm through the distal tract and then into the aortic arch through the proximal tract. Then, the hydrophilic guide wire was exchanged to stiff guide wire and the 6F right femoral sheath was exchanged to 10F sheath. Two 14-mm muscular ventricular septal defect (VSD) occluder delivery systems (jiyi VSDO, Shanghai) were advanced though the stiff guide wire and deployed at the proximal and distal narrow tract, respectively. Angiography performed 15 min later after occluders deployment showed that the aneurysm was completely isolated from the aortic cavity (Fig. 1F and G). CT angiography at 3-month follow-up confirmed the absence of contrast leakage into the aneurysm (Fig. 1H).

## 3 Discussion

Coarctation of the aorta may occur anywhere along the descending aorta and more than 95% of the cases; it is located below the origin of the LSA and may involve the origin of this vessel.^[1]^ Aneurysm may develop distal to the coarctation in patients without any intervention and is associated with significant risk of aortic rupture. Inflammation of the aortic wall, congenital weakness of the aortic arterial wall, or asymmetric and enhanced shear stress may play a significant role in the development of aneurysm formation.^[1,2]^ In the present case, the formation of the calcified biloculated aneurysm just distal to the coarctation may be associated with the relatively advanced age and the bifurcate structure composed of LSA and descending aorta. We believe that the upper portion of the aneurysm is the dilated LSA and the lower portion is the dilated descending aorta.

The management of patients presenting with complex aortic coarctation necessitates consultation of a multidisciplinary team including vascular and cardiac surgeons, interventional congenital cardiologist, and interventional radiologist. Variables that could influence treatment strategy include the degree of residual coarctation; location of the aneurysm relative to the coarctation; the shape, size, and degree of calcification of the aneurysm; suitability of landing zones for percutaneous devices deployment; patient age; and presence of collateral flow if LSA exclusion is required.^[3]^

In this case, the aortic arch aneurysm was located at the greater curvature where the LSA arose from, and was complicated by aortic arch coarctation. Conventional anatomical aortoplasty, which requires cardiopulmonary bypass or deep hypothermic circulatory arrest, would be complicated and carries a high mortality and morbidity risk.^[4]^ Endovascular approach such as percutaneous balloon dilatation and stent implantation is not suitable in cases with extensive calcification and is associated with a high procedural risk of aortic rupture. In this setting, bypass grafting between the ascending and descending aorta, instead of aortic arch reconstruction, is also effective in relieving trans-coarctation gradient and improving systemic blood perfusion. We ligated the origin of the LSA without causing cerebral ischemia or cerebral infarction because of the upper to lower flow direction. However, the aneurysm can hardly be occluded by surgical ligation. So, we intentionally constricted the proximal and distal segment of the aneurysm in order to decrease the blood flow and the pressure of the aneurysm and provide landing zones for VSD occluders, which are preconditions for the subsequent endovascular procedure of aneurysmal orifice occlusion.

## 4 Conclusion

In conclusion, the use of a 2-stage hybrid approach integrating open and endovascular repair for the treatment of a complex aortic coarctation appears feasible, safe, versatile, and effective during short-term follow-up. Besides, such a hybrid approach also offers several advantages over isolated conventional surgical repair or endovascular repair.

## Acknowledgment

The authors thank the patient participating in the present study who provided written permission for publication of this case report.
